# Impact of COVID-19 on Gender-Based Violence Prevention and Response Services in Kenya, Uganda, Nigeria, and South Africa: A Cross-Sectional Survey

**DOI:** 10.3389/fgwh.2021.780771

**Published:** 2022-01-27

**Authors:** Charlotte M. Roy, Paul Bukuluki, Sara E. Casey, Moriam O. Jagun, Neetu A. John, Nicoletta Mabhena, Mary Mwangi, Terry McGovern

**Affiliations:** ^1^Global Health Justice and Governance Program, Heilbrunn Department of Population and Family Health, Columbia University Mailman School of Public Health, New York, NY, United States; ^2^Department of Emergency Medicine, Columbia University Irving Medical Center, New York, NY, United States; ^3^Department of Social Work and Social Administration, School of Social Sciences, Makerere University, Kampala, Uganda; ^4^Center for Bridging Health Gaps, Lagos, Nigeria; ^5^ResearchLinkME, Johannesburg, South Africa; ^6^Independent Consultant, Nairobi, Kenya

**Keywords:** gender-based violence (GBV), intimate partner violence (IPV), COVID- 19, sub-Saharan Africa, emergency preparedness, gender equity

## Abstract

**Background:**

Epidemics and other complex emergencies historically have had a disproportionate impact on women and girls, increasing their vulnerability to gender-based violence (GBV). The COVID-19 pandemic has been no different, with reports of rising cases of GBV emerging worldwide. Already a significant problem in Kenya, Uganda, Nigeria, and South Africa, GBV in these countries has been exacerbated by government restrictions intended to contain the spread of COVID-19. The purpose of this study was to understand how the COVID-19 pandemic affected the availability of GBV prevention and response services from the perspective of the organizations that provide them.

**Methods:**

A cross-sectional online survey of people who work in GBV prevention and response in Kenya, Uganda, Nigeria, and South Africa was administered from July to October 2020. A convenience sample was identified through web search, contacts of in-country consultants, and relevant listservs and technical working groups. Descriptive analyses were completed using SPSS.

**Results:**

A total of 187 respondents completed the survey. Nearly all (98.9%) survey respondents reported that COVID-19 impacted their work. The majority (77.9%) stated that work decreased due to government restrictions or GBV services being deemed non-essential. The types of service most impacted were community-based prevention, shelters, and legal services. Survey respondents overwhelmingly agreed (99.3%) that COVID-19 impacted GBV prevalence and identified adolescents and women with disabilities as particularly vulnerable groups.

**Conclusions:**

GBV prevention and response services in Kenya, Uganda, Nigeria, and South Africa were highly impacted by the COVID-19 pandemic, largely due to government restrictions and the failure of governments to deem GBV services as essential. Preparedness for future crises should ensure that GBV is adequately prioritized in the initial response in order to maintain service availability with special attention paid to at-risk populations.

## Introduction

Epidemics and other complex emergencies historically have had a disproportionate impact on women and girls, increasing their vulnerability to gender-based violence (GBV). This occurs through a number of different pathways, including social isolation, economic insecurity, and the breakdown of public services (1). Exacerbating this problem, governments often de-prioritize GBV services during periods of crisis, limiting access when women and girls need them most. Increased rates of sexual violence, exploitation, and other forms of GBV were observed in the 2014–2016 Ebola outbreak in West Africa (2). GBV services in Sierra Leone, however, ceased to function almost entirely, while 80% of GBV survivors in Liberia were turned away from health clinics (3). The effect of the COVID-19 pandemic on women and girls has been similar, with reports of rising cases of GBV emerging globally.

When the World Health Organization declared COVID-19 a pandemic in March 2020, governments moved to limit the virus's spread by implementing national lockdown measures. These included curfews, movement restrictions, and public transportation shutdowns. These mandates resulted in the “Shadow Pandemic,” a surge in GBV worldwide that exposed pre-existing gender inequalities (4). The increase in GBV during COVID-19 is likely multifactorial: movement restrictions made it impossible for women to leave abusive households, while increased interpersonal and economic stress resulted in rising intimate partner violence (IPV). At the same time, GBV prevention and response services were deprioritized as governments shifted their resources to COVID-19 response, and movement regulations and curfews limited the ability of women and girls to access services.

Prior to COVID-19, GBV was a significant problem in many countries, including our study locations: Kenya, Uganda, Nigeria, and South Africa. According to Demographic and Health Surveys conducted between 2014 and 2018 in these countries, 21.3–49.9% of women reported ever experiencing physical or sexual violence by a partner (5–8). These high rates of IPV are driven by patriarchal societies that disenfranchise women politically and economically, as well as harmful gender-based practices, such as child marriage and female genital mutilation (9–11). Although these countries have made significant investments in GBV prevention and response (12, 13), GBV was largely under-prioritized even before the COVID-19 pandemic, with funding primarily coming from multilateral and bilateral donors (14). GBV response services for survivors, including health and psychosocial services, were limited and more available in urban centers. In rural areas, accessing services was a challenge, particularly due to long distances and the cost of transportation (15). Likewise, the judicial system lacked resources and capacity to deal with GBV cases adequately, and reaching legal professionals often required a long and costly trip (16). GBV prevention work was primarily supported by international non-governmental organizations (NGOs) and heavily under-funded (17).

When COVID-19 lockdown restrictions were first implemented in March 2020, the continuation of GBV services was dependent on their designation as an “essential service.” Yet, GBV prevention and response services were often not explicitly included in government plans, leading to confusion among providers. The Government of Uganda instituted one of the strictest lockdowns in the world, including the prohibition of all forms of transportation and a 7 p.m. curfew (18). It defined medicine in general as an essential service but did not release any official policy regarding GBV services. Similarly, when the Government of South Africa implemented a stay-at-home order on March 26, 2020, it made an exception for those “seeking emergency, life-saving, or chronic medical attention” (19). However, there was no specific mention of services for survivors of GBV until April 13, 2020 when President Cyril Ramaphosa issued a clarification emphasizing that GBV services must remain operational (20). The Government of Kenya did not explicitly define GBV services as essential until May 2020 (21, 22). In Nigeria, lockdown restrictions were determined by individual states, resulting in varying policies. For example, GBV was defined as essential in Abuja, whereas Lagos initially shut down their GBV response team and government-run shelters (23).

In addition to impeding access to GBV services, government-enforced movement restrictions left women trapped at home with their abusers in a context of increasing interpersonal and economic stress (1). This resulted in seemingly dramatic increases in cases of GBV. In Kenya, calls to the national GBV hotline increased by 775% in March and April 2020 (24), resulting in an additional 3,650 cases of GBV reported March through July (25). In Nigeria, government-collected data from two-thirds of the states demonstrated a 149% increase in reports of GBV from March to April, 2020 (26). In South Africa, a national counseling hotline called Lifeline SA documented a 500% increase in the number of GBV calls in the 2 months after the lockdown began (27). Meanwhile, 3,280 cases of GBV were reported to police in Uganda in April 2020 in comparison to a monthly average of 1,137 cases in 2019 (28). Although hotlines do not accurately measure GBV incidence, changes in call volume may be suggestive of changes in incidence, particularly early in a crisis when rigorous surveys are not available.

The COVID-19 pandemic had especially severe consequences for women in structurally excluded groups, such as sex workers, women with disabilities, refugees, women in rural areas and people with diverse sexual orientation, gender identity, gender expression, and sex characteristics (SOGIESC). During COVID-19, sex workers and people with diverse SOGIESC faced increased stigmatization because they were viewed as “vectors of disease,” a perception that has endured since the HIV crisis (29). This caused them to be targets of increased discrimination and violence, including targeted police brutality, and increased hesitancy among them in seeking out health care. Refugees have been hit particularly hard by the economic impacts of COVID-19, leading to increased stress and, consequently, rising incidents of GBV. For example, Yumbe, a refugee-hosting district in northwestern Uganda, has seen a 50% increase in GBV cases since the lockdown began (30).

The consequences of the COVID-19 pandemic for women and girls may take years to fully measure, but preliminary data suggests that it has significantly limited women's access to GBV services while also increasing GBV prevalence worldwide. We conducted this study to understand how the restrictions put in place by governments to contain the spread of COVID-19 affected the availability of GBV prevention and response services from the perspective of the organizations that provide them.

## Materials and Methods

We conducted a mixed methods study, including a cross-sectional online survey and in-depth interviews with GBV stakeholders in six countries. GBV stakeholders were defined as employees of organizations that work in GBV prevention and response, including community-based, national, and international NGOs, and national and local governments. This manuscript focuses on the survey data in four countries: Kenya, Uganda, Nigeria, and South Africa. Qualitative data from Kenya was published elsewhere (31); publication of qualitative findings from the other countries is forthcoming.

Survey respondents comprised a convenience sample of people who work on GBV prevention and response in the four countries. We identified respondents through a web search of organizations that address GBV, contacts of in-country consultants who work on GBV, as well as relevant listservs and technical working groups in the four countries. Web search terms included “gender-based violence,” “GBV,” “intimate partner violence,” “IPV,” “domestic violence,” and the country names. We also encouraged recipients to share the survey with peers. Efforts were made to identify respondents in different geographies and across types of GBV services (medical, psychosocial, legal, prevention) and organizations, including local and international NGOs. Anyone who participated in the survey and self-identified as working on GBV in the four countries was included.

The survey questionnaire (Supplementary Material 1) included questions on how COVID-19 affected organizations' operations and provision of services. Respondents were asked about perceived changes in the prevalence of GBV and to identify groups of women who were particularly vulnerable. We also asked respondents about changes in funding that resulted from the pandemic.

The survey was distributed via email and administered online in English using Qualtrics between July 17 and October 5, 2020. Respondents were asked to reflect on their experiences from the onset of the pandemic in March 2020 until the time when they completed the survey. Consent was obtained electronically before beginning the survey. This study was determined to be exempt by the Columbia University Institutional Review Board.

Data were cleaned and descriptive analyses performed using SPSS v27. Respondents who answered no questions beyond the demographics (22.1% of the original sample) were deleted from analysis. Responses about how COVID-19 has impacted respondents' work were collapsed from twelve into four categories to capture the main causes of changes in workload: services being deemed non-essential or government restrictions; limitations in staff, supplies, or funding; decreased use of services; and increased use of services or staff being asked to support emergency response. Five possible responses to a question about changes in the availability of specific GBV services were collapsed into three: service provision unchanged from beginning, services limited or stopped completely initially and limited or no services available now, and services stopped initially but full services available now. The original ranges of responses are shown in Supplementary Material 1.

## Results

A total of 187 respondents completed the survey: Kenya (*n* = 61), Uganda (*n* = 34), Nigeria (*n* = 64), and South Africa (*n* = 28). The majority of respondents identified their organization as a local or community-based (58.8%) or international NGO (21.3%) (Table 1). Most organizations (82.4%) addressed both GBV and sexual and reproductive health (SRH).

**Table 1:** Organization type and area of work of survey respondents.

**Characteristic**	**Kenya**	**Uganda**	**Nigeria**	**South Africa**	**Total**
	***n*** **(%)**	***n*** **(%)**	***n*** **(%)**	***n*** **(%)**	***n*** **(%)**
	**total = 61**	**total = 34**	**total = 64**	**total = 28**	**total = 187**
**Organization type(s)^a^**					
Local NGO/CBO	35 (57.4)	9 (26.5)	44 (68.8)	22 (78.6)	110 (58.8)
International NGO	17 (27.9)	8 (23.5)	12 (19.0)	2 (8.0)	39 (21.3)
International organization	5 (8.2)	4 (11.8)	5 (7.9)	1(4.0)	15 (8.2)
Government	3 (4.9)	5 (14.7)	7 (11.1)	0 (0.0)	15 (8.2)
Health facility	6 (9.8)	7 (20.6)	3 (4.8)	0 (0.0)	16 (8.7)
Shelter	0 (0.0)	2 (5.9)	1 (1.6)	3 (12.0)	6 (3.3)
Academic	1 (1.6)	2 (5.9)	1 (1.6)	0 (0.0)	4 (2.1)
**Organization sector(s)^a^**					
Clinical management	39 (63.9)	19 (55.9)	33 (51.6)	14 (50.0)	105 (56.1)
Psychosocial services	52 (85.2)	24 (70.6)	47 (73.4)	18 (64.3)	141 (75.4)
Shelter/social services	29 (47.5)	16 (47.1)	31 (48.4)	11 (39.3)	87 (46.5)
Case management	46 (75.4)	24 (70.6)	40 (62.5)	14 (50.0)	124 (66.3)
Prevention and awareness	52 (85.2)	27 (79.4)	52 (81.3)	21 (75.0)	152 (81.3)
Legal support	39 (63.9)	13 (38.2)	29 (45.3)	11 (39.3)	92 (49.2)
**Area of work**					
GBV only	9 (14.8)	6 (17.6)	10 (15.6)	8 (28.6)	33 (17.6)
GBV and SRH	52 (85.2)	28 (82.4)	54 (84.4)	20 (71.4)	154 (82.4)

a *Respondents could select more than one organization type and sector*.

### Disruptions to Organizational Operations

Nearly all respondents (98.9%) stated that COVID-19 had impacted their work. Across the four countries, 77.9% of respondents indicated that COVID-19 caused their work to stop or decrease because GBV services were deemed non-essential or due to other government restrictions (Table 2). Fewer (57.1%) respondents in South Africa reported this compared to respondents in Kenya (81.0%), Uganda (88.2%), and Nigeria (78.7%). A majority of respondents (56.4%) also stated that their work stopped or decreased due to limited supplies, staff, or funding. Few respondents (9.4%) indicated that work stopped or decreased because of decreased use of GBV services. In contrast, nearly half (47.8%) of respondents noted that their workload increased, either due to increased use of GBV services or to staff being shifted to support COVID-19 emergency response unrelated to GBV.

**Table 2:** Effect of the COVID-19 pandemic on organizational operations.

	**Kenya**	**Uganda**	**Nigeria**	**South Africa**	**Total**
	***n*** **(%)**	***n*** **(%)**	***n*** **(%)**	***n*** **(%)**	***n*** **(%)**
	**total = 61**	**total = 34**	**total = 64**	**total = 28**	**total = 187**
Work stopped or reduced due to SRH/GBV services being deemed non-essential or government restrictions	47 (81.0)	30 (88.2)	48 (78.7)	16 (57.1)	141 (77.9)
Work stopped or reduced due to limited supplies, staff, or funding	34 (58.6)	18 (52.9)	33 (54.1)	17 (60.7)	102 (56.4)
Work stopped or reduced as use of services has decreased	6 (10.3)	3 (8.8)	7 (11.5)	1 (3.6)	17 (9.4)
Workload increased due to staff being asked to support emergency response	28 (47.5)	11 (32.4)	29 (47.5)	19 (67.9)	87 (47.8)

In some regards, COVID-19 also had a positive impact on organizations in these countries by stimulating innovation. The majority of respondents (66.7%) reported observing innovative mechanisms being used to fill gaps in GBV prevention and response during COVID-19. Examples of innovative adaptations they gave included offering online counseling and referrals, establishing hotlines, and using social media and mobile apps for GBV prevention messaging.

### Interruptions by Type of Service

Some types of GBV services were more difficult to maintain under COVID-19 restrictions. Over half of respondents indicated that community-based prevention (56.6%), shelters and other social services (56.3%), and legal services (58.7%) were limited or stopped completely at the beginning of the pandemic and either remained unavailable or only partly available at the time of the survey (Table 3). The GBV service least affected by COVID-19 was clinical management of rape or other GBV, which 46.7% of respondents indicated had remained unchanged since the pandemic began. Psychosocial counseling and case management were mostly operational at the time of the survey but in a limited capacity.

**Table 3:** Changes in provision of GBV prevention and response services resulting from the COVID-19 pandemic and subsequent lockdown restrictions.

**Type of GBV service**	**Effect of COVID-19 on service**	**Kenya**	**Uganda**	**Nigeria**	**South Africa**	**Total**
	**availability**	***n*** **(%)**	***n*** **(%)**	***n*** **(%)**	***n*** **(%)**	***n*** **(%)**
Clinical management of rape or other GBV (*n* = 105)	Service provision unchanged from beginning	18 (46.2)	6 (31.6)	15 (45.5)	10 (71.4)	49 (46.7)
	Services limited or stopped completely initially and limited or no services available now	10 (25.6)	7 (36.8)	12 (36.4)	4 (28.6)	33 (31.4)
	Services stopped initially but full services available now	11 (28.2)	6 (31.6)	6 (18.2)	0 (0.0)	23 (21.9)
Counseling or psychosocial services (*n* = 141)	Service provision unchanged from beginning	18 (34.6)	7 (29.2)	11 (23.4)	10 (55.6)	46 (32.6)
	Services limited or stopped completely initially and limited or no services available now	24 (46.2)	11 (45.8)	22 (46.8)	6 (33.3)	63 (44.7)
	Services stopped initially but full services available now	10 (19.2)	6 (25.0)	14 (29.8)	2 (11.1)	32 (22.7)
Shelter and/or other social services (*n* = 87)	Service provision unchanged from beginning	9 (31.0)	3 (18.8)	5 (16.1)	9 (81.8)	26 (29.9)
	Services limited or stopped completely initially and limited or no services available now	15 (51.7)	11 (68.8)	21 (67.7)	2 (18.2)	49 (56.3)
	Services stopped initially but full services available now	5 (17.2)	2 (12.5)	5 (16.1)	0 (0.0)	12 (13.8)
Case management services (*n* = 124)	Service provision unchanged from beginning	20 (43.5)	8 (33.3)	11 (27.5)	8 (57.1)	47 (37.9)
	Services limited or stopped completely initially and limited or no services available now	18 (39.1)	12 (50.0)	18 (45.0)	6 (42.9)	54 (43.5)
	Services stopped initially but full services available now	8 (17.4)	4 (16.7)	11 (27.5)	0 (0.0)	23 (18.5)
Community-based GBV prevention (*n* = 152)	Service provision unchanged from beginning	14 (26.9)	1 (3.7)	10 (19.2)	8 (38.1)	33 (21.7)
	Services limited or stopped completely initially and limited or no services available now	30 (57.7)	18 (66.7)	29 (55.8)	9 (42.9)	86 (56.6)
	Services stopped initially but full services available now	8 (15.4)	8 (29.6)	13 (25.0)	4 (19.0)	33 (21.7)
Legal support (*n* = 92)	Service provision unchanged from beginning	15 (38.5)	2 (15.4)	6 (20.7)	4 (36.4)	27 (29.3)
	Services limited or stopped completely initially and limited or no services available now	22 (56.4)	9 (69.2)	17 (58.6)	6 (54.5)	54 (58.7)
	Services stopped initially but full services available now	2 (5.1)	2 (15.4)	6 (20.7)	1 (9.1)	11 (12.0)

### Perceived Change in Prevalence of GBV

Respondents overwhelmingly agreed (99.3%) that COVID-19 had impacted GBV prevalence in their countries. Across all four countries, the majority of respondents (90.3%) believed that GBV had increased, while a small proportion (7.6%) indicated that some forms of GBV had increased while others had decreased.

### More Severe Impact on Some Groups

Survey respondents identified several groups that had more difficulty accessing GBV services or were noted to be accessing services less during the pandemic than previously (Table 4). These groups were primarily adolescents (73.4%), followed by women with disabilities (59.1%), unmarried women (27.9%), people of diverse SOGIESC (26.6%), and migrants, refugees, and other displaced people (25.3%).

**Table 4:** Groups identified by respondents as being more severely impacted by GBV during the COVID-19 pandemic.

	**Kenya**	**Uganda**	**Nigeria**	**South Africa**	**Total**
	***n*** **(%)**	***n*** **(%)**	***n*** **(%)**	***n*** **(%)**	***n*** **(%)**
	**total = 52**	**total = 28**	**total = 53**	**total = 21**	**total = 154**
Adolescents	43 (82.7)	20 (71.4)	39 (73.6)	11 (52.4)	113 (73.4)
Women with disabilities	32 (61.5)	15 (53.6)	32 (60.4)	12 (57.1)	91 (59.1)
Ethnic minorities	10 (19.2)	1 (3.6)	9 (17.0)	5 (23.8)	25 (16.2)
Migrants, refugees, or other displaced people	9 (17.3)	10 (35.7)	13 (24.5)	7 (33.3)	39 (25.3)
People of diverse sexual orientation, gender identity	19 (36.5)	4 (14.3)	11 (20.8)	7 (33.3)	41 (26.6)
Unmarried women	15 (28.5)	5 (17.9)	20 (37.7)	3 (14.3)	43 (27.9)
Other (e.g.,: sex workers, rural women, married women, elderly women)	9 (17.3)	6 (21.4)	8 (15.1)	1 (4.8)	24 (15.6)

### Changes in Funding

When asked about changes in their organization's funding during COVID-19, a minority of survey respondents reported new or additional funding since the beginning of the pandemic from bilateral donors (29.2%), multilateral donors (18.3%), private foundations (33.3%), or community/private donors (35.1%) (Table 5). In comparison, a slightly larger proportion of respondents reported decreased funding from these sources. In particular, nearly half (41.7%) indicated that funding from their national government was reduced or stopped due to COVID-19; only 9.8% indicated that they received new or increased government funding.

**Table 5:** Changes in donor funding to respondents' organizations during COVID-19.

**Donor type**	**Change in funding**	**Kenya**	**Uganda**	**Nigeria**	**South Africa**	**Total**
		***n*** **(%)**	***n*** **(%)**	***n*** **(%)**	***n*** **(%)**	***n*** **(%)**
Bilateral donors	New/increased (*n* = 130)	14 (31.8)	7 (28.0)	13 (31.0)	4 (21.1)	38 (29.2)
	Stopped/decreased (*n* = 99)	8 (22.2)	3 (17.6)	18 (54.5)	4 (30.8)	33 (33.3)
Multilateral donors	New/increased (*n* = 115)	6 (15.8)	8 (34.8)	7 (18.4)	0 (0.0)	21 (18.3)
	Stopped/decreased (*n* = 91)	6 (19.4)	3 (18.8)	17 (54.8)	4 (30.8)	30 (33.0)
National government	New/increased (*n* = 112)	3 (7.5)	5 (26.3)	2 (5.9)	1 (5.3)	11 (9.8)
	Stopped/decreased (*n* = 75)	7 (25.9)	4 (40.0)	15 (60.0)	6 (15.4)	28 (37.3)
Private foundations	New/increased (*n* = 120)	11 (26.8)	7 (33.3)	16 (42.1)	6 (30.0)	40 (33.3)
	Stopped/decreased (*n* = 84)	6 (22.2)	4 (33.3)	21 (65.6)	4 (30.8)	35 (41.7)
Community/private donations	New/increased (*n* = 114)	6 (15.8)	8 (30.0)	19 (51.4)	9 (47.4)	40 (35.1)
	Stopped/decreased (*n* = 87)	9 (32.1)	4 (30.8)	19 (63.3)	3 (18.8)	35 (40.2)

Respondents in Nigeria more often reported funding cuts compared to those in Kenya, Uganda, and South Africa. For example, 65.6% of survey respondents in Nigeria indicated that funding from private foundations had been reduced or stopped due to COVID-19, while only 22.2% of respondents in Kenya, 30.8% in South Africa, and 33.3% in Uganda reported decreased funding from private foundations.

## Discussion

Our findings suggest that organizations addressing GBV prevention and response in Kenya, Uganda, Nigeria, and South Africa were highly impacted by the COVID-19 pandemic. Across countries and types of organizations, respondents reported a negative impact that was largely attributed to government restrictions and the failure of governments to clearly deem GBV services as “essential” in the early lockdown period. The majority of respondents indicated that limitations related to staff, supplies, and funding impeded their ability to continue providing services. For example, movement and travel restrictions within and across countries limited the movement of staff and supplies, leading to shortages. These findings reflect the de-prioritization of GBV services during the pandemic. In these countries, where GBV is endemic and services were under-resourced even prior to the pandemic, this will have devastating long-term consequences for women and girls.

Respondents indicated that community-based prevention services were most disrupted by the pandemic, with the majority reporting that GBV prevention activities still remained limited or unavailable entirely. Prevention activities were likely most impacted because they typically rely on face-to-face interaction, often in large groups. For GBV prevention programming that builds community awareness and transformation over the course of years, this may result in the loss of prior gains or momentum. At the same time, this forced change pushed organizations to develop innovative approaches to GBV prevention. For example, the Ugandan NGO Raising Voices recommended adapting SASA! community mobilization programming by moving discussions to virtual platforms such as WhatsApp, disseminating paper copies of activities for families, and using radio to air SASA! “soap operas” (32). Such innovations could increase programs' reach in the future, though the ability of poor, rural women and girls to engage in web- or phone-based programs remains a major constraint (33).

Among GBV response services, respondents indicated that shelters and legal services were the most likely to remain limited or unavailable at the time of the survey. Shelters in particular faced challenges in adapting to new requirements for social distancing and testing (34). Yet, many organizations found innovative ways to continue their GBV response activities despite COVID-19 restrictions. For example, FIDA-Kenya launched a toll-free hotline to facilitate the provision of legal and psychosocial support services while transportation restrictions were in place (35). In South Africa, staff at People Opposing Women Abuse continued to coordinate GBV case management using WhatsApp and provided psychosocial and counseling services via phone and social media (36).

Interestingly, a smaller proportion of respondents from South Africa indicated that their work had been stopped or reduced due to government restrictions or GBV services being deemed non-essential compared to other countries in the survey. In addition, a greater proportion of respondents from South Africa reported the provision of response services, including clinical management, psychosocial services, case management, and shelters, were unchanged since the beginning of the pandemic. This is likely because, relative to the other countries, South Africa was quicker to specify that GBV response services must remain open during the government lockdown (20). This reflects the importance of a government's early prioritization of GBV prevention and response to maintaining service provision during a pandemic or other complex emergency.

Survey findings related to respondents' perceptions of the change in the prevalence of GBV during the pandemic support the documented rise in GBV cases in the four countries, also found in our in-depth interviews with GBV service providers and donors (31). Respondents also identified a number of vulnerable groups that were more severely impacted by increased GBV prevalence, in particular adolescents and women with disabilities. School closures have resulted in adolescents spending more time in the community where they are vulnerable to sexual exploitation and abuse, while also cutting them off from a vital source of information and support (37). Meanwhile, women with disabilities, who face increased rates of GBV at baseline, have been rendered more vulnerable during the pandemic by the lack of accessible communications for the vision- and hearing-impaired and transportation shutdowns that increased the barriers to accessing services (26). Preparedness for future crises should ensure that GBV is adequately prioritized in the initial response in order to maintain service availability with special attention paid to these at-risk populations.

Reported changes in funding during COVID-19 varied across organizations, with some reporting that they received additional funding while others stated that they lost funding. Of note, very few respondents indicated that they were given any additional funding from the government, while nearly half stated that their government funding was cut. Once again, this suggests a de-prioritization of GBV prevention and response by the government, even as cases of GBV rose dramatically. Further research on shifts in funding due to COVID-19 may help to understand overall changes in funding and how losses in funding from some groups may have been offset by gains from others.

Overall, our findings show that the GBV organizations surveyed struggled to remain operational during COVID-19. This reflects the lack of resources and funding dedicated to addressing GBV that existed even prior to the pandemic (3). The COVID-19 pandemic did not create new inequalities; rather, it unveiled the disparities and de-prioritization of the well-being of women and girls that have long pervaded many societies around the world. Preparedness for future epidemics must integrate interventions that promote gender equity into emergency response. This includes clearly defining comprehensive GBV services as essential so that they continue operating during lockdowns and ensuring that women and girls are able to access these services. Individual organizations also must create preparedness plans for how they will continue to provide GBV prevention and response services when physical accessibility and staff are limited.

One limitation of this study is that many respondents did not complete the full survey, which resulted in decreasing sample sizes for later survey questions. In addition, we attempted to obtain a broad sample of GBV organizations in the four countries; however the sample skewed toward local and international NGOs with less robust representation from government organizations. The study used a convenience sample that may not have been representative of all organizations working in GBV prevention and response in these countries. For example, organizations with less internet presence or who did not participate in technical working groups were less likely to receive the survey invitation. Furthermore, our findings may under-represent the challenges faced by organizations since the groups most impacted by the COVID-19 pandemic may not have had time to participate. In contrast, organizations less impacted by COVID-19 may not have felt as compelled to participate.

## Data Availability Statement

The raw data supporting the conclusions of this article will be made available by the authors, without undue reservation.

## Ethics Statement

This study was reviewed and approved by the Columbia University IRB. The participants provided their written informed consent to participate in this study.

## Author Contributions

SC, NJ, and TM participated in the study conception, design, and implementation. SC, NJ, PB, MJ, NM, and MM implemented the study. CR, SC, and NJ participated in analysis of the data. CR drafted the manuscript. NJ, SC, and TM contributed to the writing process. All authors contributed to the article and approved the submitted version.

## Funding

This study was funded by Ford Foundation (No. 133187).

## Conflict of Interest

The authors declare that the research was conducted in the absence of any commercial or financial relationships that could be construed as a potential conflict of interest.

## Publisher's Note

All claims expressed in this article are solely those of the authors and do not necessarily represent those of their affiliated organizations, or those of the publisher, the editors and the reviewers. Any product that may be evaluated in this article, or claim that may be made by its manufacturer, is not guaranteed or endorsed by the publisher.
