# Supplementary material for: Impact of COVID-19 on Gender-Based Violence Prevention and Response Services in Kenya, Uganda, Nigeria, and South Africa: A Cross-Sectional Survey
Source: Front Glob Womens Health. 2022 Jan 27;2:780771. doi: 10.3389/fgwh.2021.780771 (PMC8829509; doi:10.3389/fgwh.2021.780771)
Supplement: Supplementary file 1 [file Data_Sheet_1.PDF]

Supplementary material 1. GBV survey tool

Please read the following information and click “I agree” if you would like to continue.
You are invited to participate in this study on the impact of COVID-19 on GBV/SRH services, prevention, and funding. The project is being conducted by the Program on Global Health Justice and Governance (PGHJG) at Columbia University Mailman School of Public Health based in New York, USA. In this study, we are seeking to gain information on any shifts in policies, service provision, and demand of GBV and SRH services and products due to COVID-19. We hope this study will contribute to future responses to GBV and SRH in emergencies and inform policy recommendations regarding how to implement essential GBV and SRH services within emergency preparedness in future pandemics. This study also seeks to identify and promote funding practices that reinforce effective GBV and SRHR responses during emergency responses. If you agree to participate, you will complete the online survey, which will take approximately 15-20 minutes. If you have worked in multiple countries, we ask that you repeat the survey for each country. You will be asked questions relating to your work in gender-based violence and sexual and reproductive health, as well as the state of GBV/SRH services and prevention more broadly in the country in the wake of COVID-19. The data will be stored on a password protected computer with no mention of your name. The data will be destroyed once the study is completed. We will not collect any personal, private, or identifying information. All responses will be kept confidential.

The only known risk to you of your involvement in this study is the inconvenience of giving your time. There are no direct benefits to you for participating in this discussion, but we hope to inform policy around implementing essential GBV and SRH services and funding within emergency preparedness in future pandemics.

If you have questions about the study, please contact Neetu John at neetu.john@columbia.edu.

If you consent to participate in this study, please select agree to begin the survey:

- ○ I agree
- ○ I disagree

E1 Which country are you working in?

- ◯ Colombia
- ◯ Kenya
- ◯ Nigeria
- ◯ South Africa
- ◯ Uganda
- ◯ USA
- ◯ Other [Please Elaborate] ____

Skip To: E2 If Which country are you working in? = USA
Skip To: E3 If Which country are you working in? = Colombia
Skip To: E3 If Which country are you working in? = Kenya
Skip To: E3 If Which country are you working in? = Nigeria
Skip To: E3 If Which country are you working in? = South Africa
Skip To: E3 If Which country are you working in? = Uganda

E2 Which state do you primarily work in?

- ◯ Louisiana
- ◯ Maine
- ◯ Minnesota
- ◯ New Mexico
- ◯ New York
- ◯ North Carolina
- ◯ South Dakota
- ◯ Texas
- ◯ Washington
- ◯ Wisconsin
- ◯ Other [Please Elaborate] ____

E3 What kind of organization do you work for? **[Please select all that apply]**

- ☐ Local NGO
- ☐ International NGO
- ☐ International Organization (for example, UN agencies, World Bank, etc)
- ☐ Government
- ☐ Community-based Organization
- ☐ Health facility (for example, Clinic, health center, hospital, etc)
- ☐ Shelter
- ☐ Other [Please Elaborate] ________________

E4 What is your area of work?

- ○ Gender-based violence (GBV)
- ○ Sexual and reproductive health (SRH)
- ○ Both GBV and SRH
- ○ We do not work on either GBV or SRH

Skip To: End of Survey If What is your area of work? = We do not work on either GBV or SRH

E5 What age group(s) does your organization serve? **[Please select all that apply]**

- ☐ Children (0-9 years)
- ☐ Very young adolescents (10-14 years)
- ☐ Adolescents (15-19 years)
- ☐ Adults (18 years or older)
- ☐ Any ages

E6 How would you best describe your primary role?

- ○ Program manager
- ○ Health worker (for example, nurse, midwife, doctor, etc)
- ○ Social worker
- ○ Community outreach worker / Community health worker
- ○ Educator/ Trainer
- ○ Other [Please elaborate] ____

E7 Has COVID-19 impacted your work or changed how you work?

- ○ Yes
- ○ No

Skip To: E14 If Has COVID-19 impacted your work or changed how you work? = No

E8 How has COVID-19 impacted your work at any point since the pandemic began? [Please select all that apply]

- ☐ Work has stopped/reduced as SRH and/or GBV clinics/services were deemed non-essential by the government
- ☐ Work was stopped/reduced due to lockdown/movement restrictions that have been imposed since the beginning of the pandemic
- ☐ Work was stopped initially due to lockdown/movement restrictions imposed in the beginning of the pandemic, but have since resumed after restrictions were reduced/lifted
- ☐ Work has stopped/reduced as supplies and commodities are unavailable or limited in supply (for example, due to difficulties with procurement and transport)
- ☐ Work has stopped/reduced as we do not have sufficient personal protective gear and other infection management supplies
- ☐ Work has stopped/reduced as our staff have been diverted to emergency response
- ☐ Work has stopped/reduced as our funding was diverted to emergency response
- ☐ Work has stopped/reduced as we do not have the capacity and resources to switch to remote work
- ☐ Work has reduced as use or demand for services has reduced
- ☐ Work has reduced as only some work can be done remotely
- ☐ Workload has increased as we are being asked to support the emergency response in addition to our regular work
- ☐ Workload has increased as use and demand for services has increased
- ☐ We are trying innovative ways to continue service delivery using technology and/or other strategies

☐ Other [Please elaborate] ________________________________

E9 What gender-based violence (GBV) services does your organization provide and which ones have you limited or stopped since the beginning* of COVID-19? [Please select all that apply] (*by beginning we mean when lockdowns and other restrictive policies were imposed)

	Service provision unchanged from the beginning	Limited service provided from beginning	Service stopped completely from beginning	Services stopped initially but full services available now	Services stopped initially but limited services available now	Not applicable/ we do not provide this service
Clinical management of rape or other GBV	○	○	○	○	○	○
GBV Counselling or psychosocial services	○	○	○	○	○	○
GBV Shelter and/or other social services	○	○	○	○	○	○
GBV case management services	○	○	○	○	○	○
Community-based GBV prevention/GBV awareness-raising activities	○	○	○	○	○	○
Legal support for GBV survivors	○	○	○	○	○	○
Other (Please elaborate)	○	○	○	○	○	○

E10 What sexual and reproductive health (SRH) services does your organization provide and which ones have you limited or stopped since the beginning* of COVID-19? [Please select all that apply]
(*by beginning we mean when lockdowns and other restrictive policies were imposed)

	Service provision unchange d from the beginning	Limited service provided from beginnin g	Service stopped completel y from beginning	Services stopped initially but full services availabl e now	Services stopped initially but limited services availabl e now	Not applicable / we do not provide this service
Contraceptive counselling and services	○	○	○	○	○	○
Abortion care/ post abortion care	○	○	○	○	○	○
Delivery care	○	○	○	○	○	○
Antenatal/postnata l care	○	○	○	○	○	○
STI screening and treatment	○	○	○	○	○	○
HIV testing, care and treatment	○	○	○	○	○	○
Reproductive cancer screening	○	○	○	○	○	○
Adolescent-friendly or focused SRH services	○	○	○	○	○	○
Other SRH Clinical services (Please elaborate)	○	○	○	○	○	○
Community outreach and awareness related to SRH	○	○	○	○	○	○
Other [please elaborate]	○	○	○	○	○	○

E11 Do you see that any particular groups of women are having more trouble accessing your GBV or SRH services or are accessing these services less during the pandemic?

- ◯ Yes
- ◯ No
- ◯ Don’t know

Skip To: E14 If Do you see that any particular groups of women are having more trouble accessing your GBV or SRH... = No

Skip To: E14 If Do you see that any particular groups of women are having more trouble accessing your GBV or SRH... = Don’t know

E12 Which groups of women are having more trouble accessing your GBV or SRH services or accessing them less during the pandemic? [Please select all that apply]

- ▢ Adolescents
- ▢ Women with disabilities
- ▢ Ethnic minorities
- ▢ Migrants, refugees or other displaced people
- ▢ People of diverse sexual orientation, gender identity and expression or sex characteristics
- ▢ Unmarried women
- ▢ Other [Please elaborate] ____

E13 Has your organization utilized any innovative mechanisms to fill some of the gaps in GBV/SRH services and prevention efforts?

- ◯ Yes
- ◯ No
- ◯ Don’t know

Skip To: E15 If Has your organization utilized any innovative mechanisms to fill some of the gaps in GBV/SRH serv... = No

Skip To: E15 If Has your organization utilized any innovative mechanisms to fill some of the gaps in GBV/SRH serv... = Don’t know

E14 Please provide an example of how your organization has utilized innovative mechanisms to fill some of the gaps in GBV/SRH services and prevention efforts.

____

E15 Are you or members of your organization involved in any local, regional or national taskforce on COVID-19 response and recovery?

- ◯ Yes
- ◯ No
- ◯ Don’t Know

E16 To support your COVID-19 (GBV and/or SRH) response has your organization received new or additional funding from: **[**Please select all that apply]

	YES	NO	NA
Bi-lateral donors	○	○	○
Multi-lateral donors	○	○	○
National Government	○	○	○
Private foundations	○	○	○
Community/private donations	○	○	○
Other (Please elaborate)	○	○	○

E17 As a result of COVID-19 has (GBV and/or SRH) funding to your organization been reduced or stopped from: **[**Please select all that apply]

	YES	NO	NA
Bi-lateral donors	○	○	○
Multi-lateral donors	○	○	○
Private foundations	○	○	○
National Government	○	○	○
Community/private donations	○	○	○
Other (Please elaborate)	○	○	○

E18 What else are current funders doing to support your organization’s effort to respond to the impact of COVID-19? **[Please select all that apply]**

	YES	NO
Converting restricted grant to unrestricted funding	○	○
Accelerating payment schedules on grants	○	○
Extending the timeframe of current grant(s) without penalty	○	○
Allowing goals of current grant(s) to shift	○	○
Waiving or making reporting deadlines flexible	○	○
Communicating one-on-one with you about the effect of COVID-19 on your organization	○	○
Other (Please elaborate)	○	○

E19 The following questions pertain to general GBV and SRH service provision in this country (not limited to your organization).

Has COVID-19 impacted GBV service provision and prevention in this country?

○ Yes

○ No

○ I don’t know / not my area of expertise

Skip To: E24 If The following questions pertain to general GBV and SRH service provision in this country (not lim... = No

Skip To: E29 If The following questions pertain to general GBV and SRH service provision in this country (not lim... = I don’t know / not my area of expertise

E20 How has COVID-19 impacted GBV service provision and prevention? **[**Please select all that apply]

- ☐ Full on-site services are available but there has been a reduction in use or demand
- ☐ Full on-site services are available but there has been an increase in use or demand
- ☐ Services have stopped as GBV clinics/services were deemed non-essential by the government
- ☐ Services have stopped/reduced since the beginning of the pandemic due to lockdown/movement restrictions imposed in the country
- ☐ Services were stopped initially due to lockdown/movement restrictions imposed in the beginning of the pandemic, but have since resumed after restrictions were reduced/lifted
- ☐ Services have stopped/reduced as staff have been diverted to emergency response
- ☐ Services have stopped/reduced as funding has been diverted to emergency response
- ☐ Services have stopped/reduced as there are shortages of personal protective gear and infection management supplies
- ☐ Services have been reduced as use or demand has decreased
- ☐ Services have reduced as staff have additional COVID-19 responsibilities
- ☐ Services have reduced as essential supplies and commodities are in short supply (for example, due to difficulties with procurement and transport)
- ☐ Service delivery has shifted to remote provision via technology or other innovative strategies

☐ Other [Please elaborate]

E21 What type of GBV services have been limited or stopped since the beginning* of COVID-19?   [Please select all that apply]   (* by beginning we mean when lockdowns or other restrictive policies were imposed)?

	Service provision unchanged from beginning	Limited service provided from beginning	Service stopped completely from beginning	Services stopped initially but full services available now	Services stopped initially but limited services available now	Not applicable/ we do not provide this services
Clinical management of rape or other GBV	○	○	○	○	○	○
Counselling or psychosocial services	○	○	○	○	○	○
Shelter and/or other social services	○	○	○	○	○	○
GBV case management services	○	○	○	○	○	○
Community-based GBV prevention/GBV awareness-raising activities	○	○	○	○	○	○
Legal support for GBV survivors	○	○	○	○	○	○
Police services for GBV survivors	○	○	○	○	○	○
Judicial services for GBV survivors	○	○	○	○	○	○
Other [Please elaborate]	○	○	○	○	○	○

E22 Do you see that any particular groups of women are having more trouble accessing services or are accessing GBV services less during the pandemic in this country?

- ◯ Yes
- ◯ No
- ◯ Don’t know

Skip To: E24 If Do you see that any particular groups of women are having more trouble accessing services or are... = No

Skip To: E24 If Do you see that any particular groups of women are having more trouble accessing services or are... = Don’t know

E23 Which groups of women are having more trouble accessing services or are accessing GBV services less during the pandemic in this country? [Please select all that apply]

- ▢ Adolescents
- ▢ Women with disabilities
- ▢ Ethnic minorities
- ▢ Migrants, refugees or other displaced people
- ▢ People of diverse sexual orientation, gender identity and expression or sex characteristics
- ▢ Unmarried women
- ▢ Other [Please elaborate] ____

E24 Do you think that COVID-19 is impacting the prevalence of GBV and/or intimate partner violence (IPV) in this country?

- ◯ Yes
- ◯ No
- ◯ Don't know

Skip To: E27 If Do you think that COVID-19 is impacting the prevalence of GBV and/or intimate partner violence (I... = No

Skip To: E27 If Do you think that COVID-19 is impacting the prevalence of GBV and/or intimate partner violence (I... = Don't know

---

E25 How has COVID-19 affected IPV/GBV prevalence in this country?

- ◯ Reports of GBV/IPV have increased
- ◯ Reports of GBV/IPV have reduced
- ◯ Reports of some forms of GBV (such as IPV) have increased, but other forms (such as non-partner violence) have reduced [Please elaborate] ____
- ◯ Other [Please elaborate] ____

E26 How did you make these conclusions about the changes in GBV prevalence in this country? **[**Please select all that apply]

- ☐ News/media reports
- ☐ Social media groups
- ☐ Administrative statistics provided by the government
- ☐ Direct reports from women affected
- ☐ Changes in demand for GBV services as indicated by GBV services delivery data
- ☐ Internal organization communications
- ☐ Communications with professional networks
- ☐ Other [Please elaborate]

____

E27 Have you seen examples of innovative mechanisms being effectively used to fill some of the gaps in GBV prevention and response in this country?

- ○ Yes
- ○ No
- ○ Don’t know

Skip To: E29 If Have you seen examples of innovative mechanisms being effectively used to fill some of the gaps i... = No

Skip To: E29 If Have you seen examples of innovative mechanisms being effectively used to fill some of the gaps i... = Don’t know

E28 Please provide an example of innovative mechanisms being effectively used to fill some of the gaps in GBV prevention and response in this country.

____

E29 Has the COVID-19 pandemic impacted the provision of sexual and reproductive health (SRH) services in this country?

- ◯ Yes
- ◯ No
- ◯ I don’t know/ not my area of expertise

Skip To: E34 If Has the COVID-19 pandemic impacted the provision of sexual and reproductive health (SRH) services... = No

Skip To: E34 If Has the COVID-19 pandemic impacted the provision of sexual and reproductive health (SRH) services... = I don’t know/ not my area of expertise

E30 How has COVID-19 impacted SRH service provision and outreach? [Please select all that apply]

- ☐ Full on-site services are available but there has been a reduction in use or demand
- ☐ Full on-site services are available but there has been an increase in use or demand
- ☐ All services have stopped due to lockdown/movement restriction
- ☐ Services have stopped as SRH clinics/services were deemed non-essential by the government
- ☐ Services have stopped/reduced since the beginning of the pandemic due to lockdown/movement restrictions imposed in the country
- ☐ Services were stopped initially due to lockdown/movement restrictions imposed in the beginning of the pandemic, but have since resumed after restrictions were reduced/lifted
- ☐ Services have stopped/reduced as staff have been diverted to emergency response
- ☐ Services have stopped/reduced as funding was diverted to emergency response
- ☐ Services have stopped/reduced as there are shortages of personal protective gear and infection management supplies
- ☐ Services have been reduced as use or demand has decreased
- ☐ Services have reduced as staff have additional COVID-19 responsibilities
- ☐ Services have reduced due to reduced availability of supplies and commodities (for example, due to difficulties with procurement and transport)

☐ Service delivery has shifted to remote provision via telemedicine or other innovative strategies

☐ Other (please elaborate) ____

E31 What type of SRH services have been limited or stopped since the beginning* of COVID-19?**[**Please select all that apply]
(* by beginning we mean when lockdowns or other restrictive policies were imposed)

	Service provision unchanged from beginning	Limited service provided from beginning	Service stopped completely from beginning	Services stopped initially but full services available now	Services stopped initially but limited services available now	Not applicable / we do not provide this service
Contraceptive counselling and services	○	○	○	○	○	○
Abortion/ post abortion care	○	○	○	○	○	○
Delivery care	○	○	○	○	○	○
Antenatal/postnatal care	○	○	○	○	○	○
STI screening and treatment	○	○	○	○	○	○
HIV testing, care and treatment	○	○	○	○	○	○
Reproductive cancer screening	○	○	○	○	○	○
Adolescent-friendly or focused SRH services	○	○	○	○	○	○
Other SRH Clinical services (Please elaborate)	○	○	○	○	○	○
Community outreach and awareness related to SRH	○	○	○	○	○	○
Other (Please elaborate)	○	○	○	○	○	○

E32 Do you see that any particular groups of women are having more trouble accessing services or are accessing SRH services less during the pandemic in this country?

◯ Yes

◯ No

◯ Don’t know

Skip To: E34 If Do you see that any particular groups of women are having more trouble accessing services or are... = No

Skip To: E34 If Do you see that any particular groups of women are having more trouble accessing services or are... = Don’t know

E33 Which groups of women are having more trouble accessing services or are accessing SRH services less during the pandemic in this country? **[**Please select all that apply]

▢ Adolescents

▢ Women with disabilities

▢ Ethnic minorities

▢ Migrants, refugees or other displaced people

▢ People of diverse sexual orientation, gender identity and expression or sex characteristics

▢ Unmarried women

▢ Other [Please elaborate] ____

E34 Have you heard that because of Covid-19 women are having difficulty obtaining contraceptives or continuing contraceptive use in this country?

- ◯ Yes
- ◯ No
- ◯ Don’t know

Skip To: E36 If Have you heard that because of Covid-19 women are having difficulty obtaining contraceptives or c... = No

Skip To: E36 If Have you heard that because of Covid-19 women are having difficulty obtaining contraceptives or c... = Don’t know

E35 How did you make these conclusions about women’s use of contraception and/or abortion in this country? [Please select all that apply]

- ☐ News/media reports
- ☐ Social media groups
- ☐ Administrative statistics provided by the government
- ☐ Direct reports from women affected
- ☐ Changes in demand for our SRH services as indicated by SRH service delivery data
- ☐ Internal organization communications
- ☐ Professional Networks within the field
- ☐ Other (please elaborate) ____

E36 Have you heard that because of Covid-19 women are having difficulty obtaining a safe abortion in this country?

○ Yes

○ No

○ Don’t know

Skip To: E38 If Have you heard that because of Covid-19 women are having difficulty obtaining a safe abortion in... = No

Skip To: E38 If Have you heard that because of Covid-19 women are having difficulty obtaining a safe abortion in... = Don’t know

E37 How did you make these conclusions about women’s use of contraception and/or abortion in this country? **[**Please select all that apply]

☐ News/media reports

☐ Social media groups

☐ Administrative statistics provided by the government

☐ Direct reports from women affected

☐ Changes in demand for our SRH services as indicated by SRH service delivery data

☐ Internal organization communications

☐ Professional Networks within the field

☐ Other (please elaborate) ____

E38 Have you seen innovation and technology being used effectively to cover some of the gaps in SRH service provision in this country?

- ◯ Yes
- ◯ No
- ◯ Don’t know

E39 Please provide an example of innovation and technology being used effectively to cover some of the gaps in SRH service provision in this country.

____
